# Concurrent thyroid ima artery, high-riding brachiocephalic trunk, and supreme intercostal artery in thoracic vertebral artery configuration: a computed tomography angiography case report

**DOI:** 10.1007/s00276-026-03924-9

**Published:** 2026-06-15

**Authors:** Mugurel Constantin Rusu

**Affiliations:** Division of Anatomy, Faculty of Dentistry, “Carol Davila” University of Medicine and Pharmacy, 050474 Bucharest, Romania

**Keywords:** Thyroid ima artery, Brachiocephalic trunk, Thoracic vertebral artery, Supreme intercostal artery, Computed tomography angiography, Anatomical variation

## Abstract

**Purpose:**

To report a cluster of five concurrent cervical vascular variants identified on computed tomography angiography (CTA), including a thyroid ima artery (TIA) from a high-riding brachiocephalic trunk (BCT), a supreme intercostal artery (SIA) in thoracic vertebral artery (TVA) configuration, and associated variants, and to discuss their embryological basis and combined surgical implications.

**Methods:**

Retrospective review of an archived CTA study of a 74-year-old female patient, using the Horos DICOM workstation with multiplanar reformatting and three-dimensional volume-rendered reconstruction. All measurements were performed by a single observer, repeated on a separate session, and the mean reported.

**Results:**

Five concurrent vascular variants and one skeletal variant were identified. (1) A common origin of the brachiocephalic trunk and left common carotid artery (the so-called bovine aortic arch). (2) A high pretracheal BCT course (calibre 1.29 cm, length 2.9 cm) with its upper margin 1.74 cm above the manubrial notch, crossing the anterior trachea from left to right. (3) A TIA arising from the BCT immediately proximal to its bifurcation and supplying both thyroid lobes. (4) An inferior mediastinal loop of the right subclavian artery (1.72 cm) with an intrathoracic origin of the right vertebral artery at 0.99 cm below the neck of the first rib. (5) A right SIA (calibre 1.1 mm) arising from the right vertebral artery at the level of the C7 transverse process in the TVA configuration, passing through the C7 transverse foramen and descending posterior to the necks of ribs 1–3. In addition, bilateral poor sternoclavicular joint articulation converted the suprasternal notch into an interclavicular space simultaneously containing the BCT, TIA, and thyroid isthmus.

**Conclusion:**

This combination of variants places multiple vessels in a shared pretracheal/interclavicular operative plane and appears to be undocumented as a combined pattern in the anatomical literature reviewed for this report. The embryological basis, clinical implications, and role of preoperative CTA in detection are discussed.

## Introduction

The thyroid ima artery (TIA), also known as the arteria thyroidea ima or the artery of Neubauer, is an inconstant unpaired vessel that ascends anterior to the trachea to supply the thyroid isthmus and inferior poles. Its pooled prevalence in adults is approximately 3.3–3.8%, with the brachiocephalic trunk (BCT) as the most frequent origin (~ 74% of TIA cases) [2, 11]. A high-lying (high-riding) BCT is defined by Cai et al. [3] either as its upper margin crossing the anterior tracheal midline 2 cm or more above the suprasternal notch, or as a course reaching above the sixth tracheal cartilage; the former criterion is met in approximately 2.2% of surgical candidates. A BCT arising from a common origin with the left common carotid artery may follow a high pretracheal course and may coexist with a TIA arising from it [3, 9]. When present together, these variants place two high-pressure vessels anterior to the trachea during tracheotomy, thyroidectomy, and emergency airway procedures.

The supreme intercostal artery (SIA) normally arises from the costocervical trunk of the subclavian artery. An anomalous origin from the vertebral artery, in the so-called thoracic vertebral artery (TVA) configuration, is recognised in anatomical reference texts [1] and has been documented angiographically [5–7], but remains a rare imaging finding. We present a case in which these three variants co-occurred with additional posterior thoracic and skeletal findings, all characterised on CTA. To our knowledge, and within the literature reviewed for this report, this particular combination has not previously been described.

## Case report

An archived CTA study of a 74-year-old female was reviewed on the Horos DICOM workstation (open-source) with multiplanar reformatting and three-dimensional reconstruction. The study had been acquired on a 64-slice Siemens SOMATOM Definition AS scanner (Siemens Healthineers, Forchheim, Germany) at 120 kVp with automatic tube-current modulation (CARE Dose4D; 150–200 reference mAs), a 64 × 0.6 mm detector configuration with z-flying focal spot, gantry rotation time 0.5 s, and pitch 0.9. Iodinated contrast medium (iohexol 350 mg I/mL, 70 mL) was injected intravenously at 4 mL/s followed by a 30 mL saline chaser, with bolus tracking in the ascending aorta (threshold 100 HU). Axial images were reconstructed at 0.75 mm slice thickness with 0.5 mm increment using a medium-smooth vascular kernel (B26f). All measurements were performed directly on the DICOM images by a single observer using the built-in caliper tool; each measurement was repeated on a separate session and the mean value is reported. Formal inter-observer reliability testing was not undertaken, as a single reader reviewed the study; this is acknowledged as a limitation. No other clinical data are available for this retrospective imaging review.

The aortic arch showed a common origin of the brachiocephalic trunk and the left common carotid artery (L-CCA)—the so-called bovine aortic arch, a colloquial term whose use is discouraged in formal anatomical description—with both vessels sharing a common trunk (calibre 2.03 cm, length 0.94 cm) arising at the level of the T1–T2 intervertebral disc. The BCT (calibre 1.29 cm, length 2.9 cm) originated from this common trunk 1.74 cm postero-superiorly to the superior margin of the manubrium sterni and crossed the anterior trachea from left to right above the sternal manubrium, immediately inferior to the thyroid gland, before dividing into the right common carotid and right subclavian arteries posterior to the right sternoclavicular joint. Its upper margin lay 1.74 cm above the manubrial notch. The left-to-right tracheal crossing course, a consequence of the bovine arch origin, expanded the zone of anterior tracheal contact compared with a normally right-sided BCT.

Immediately proximal to the BCT bifurcation, a TIA arose and coursed with an inferior loop from right to left across the anterior BCT surface, lying postero-superiorly to the superior margin of the manubrium sterni. The TIA lay 0.49 cm from the right thyroid lobe and 0.77 cm from the isthmus. Its main vessel reached the inferior pole of the left thyroid lobe, while a subsidiary branch supplied the right lobe and isthmus, demonstrating bilateral distribution.

After the BCT bifurcated, the right subclavian artery (RSA) looped inferiorly 1.72 cm into the upper mediastinum on the right side of the trachea, contacting the medial face of the right lung apex. The right vertebral artery (calibre 2.7 mm) arose from the ascending arm of this RSA loop, 0.99 cm below the neck of the first rib, yielding an entirely intrathoracic initial segment of the right vertebral artery.

From the right vertebral artery, at the level of the C7 transverse process, a SIA (calibre 1.1 mm) arose. Its initial segment measured 8.9 mm and was directed posteriorly. The artery passed through the transverse foramen of the seventh cervical vertebra and then descended posterior to the necks of ribs 1–3 and anterior to their respective transverse processes.

Both thyroid lobes extended posterior to the upper segments of the medial clavicular extremities. The medial clavicular extremities demonstrated poor sternoclavicular joint (SCJ) articulation bilaterally, defined as less than 25% of the clavicular cortical surface articulating with the manubrium [10], a finding present in 4.8% of asymptomatic individuals on chest CT. As a consequence of this poor articulation, combined with the posterior extension of the thyroid lobes behind the clavicular heads, the suprasternal notch took on the character of an interclavicular space. This space, anterior to the trachea, simultaneously contained the high-coursing BCT, the TIA, and the thyroid isthmus.

**Fig. 1:**
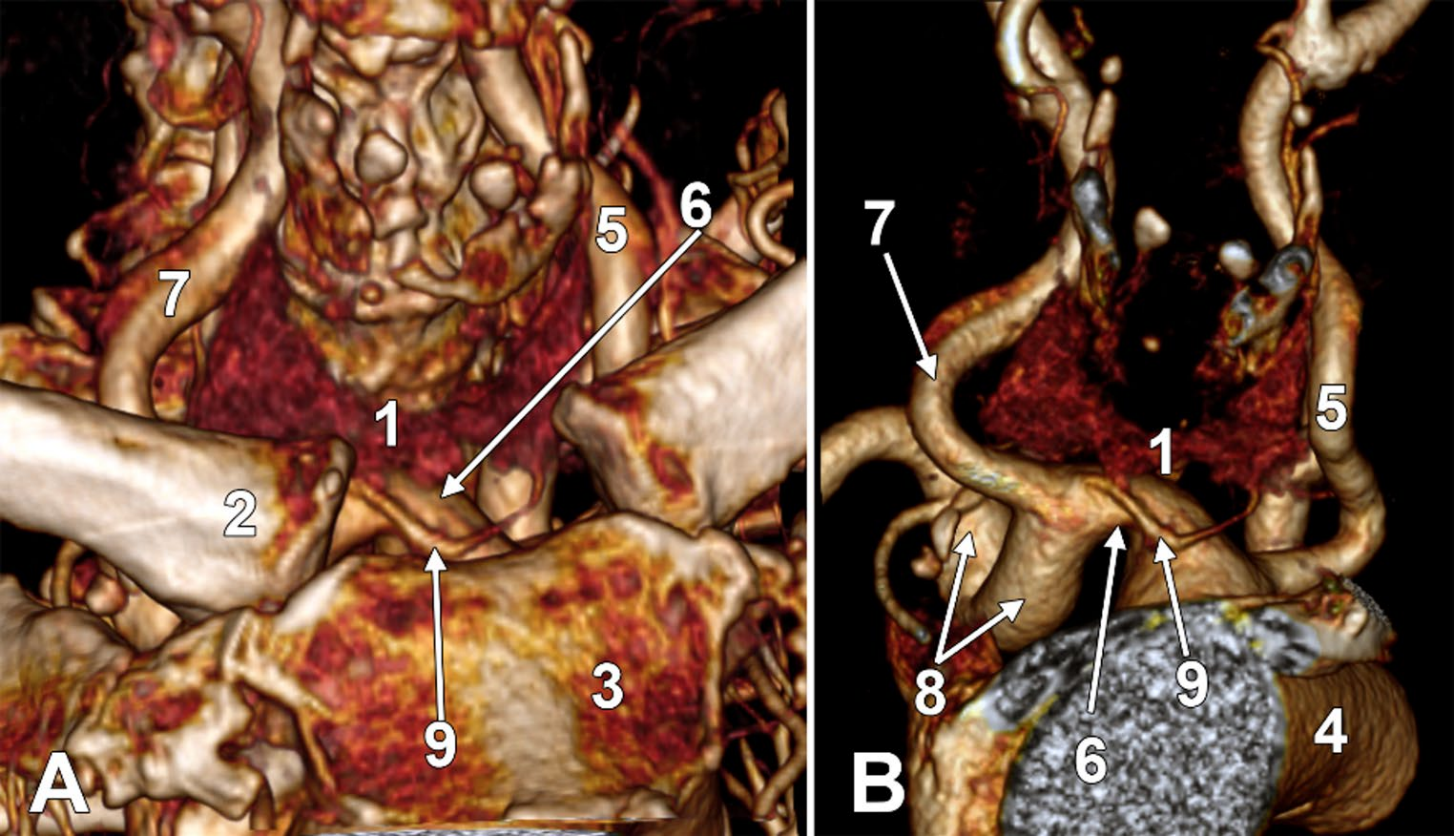
Three-dimensional CTA reconstructions (Horos, A and B: anterior views). **1** thyroid isthmus; **2** sternal end of the right clavicle; **3** sternal manubrium; **4** aortic arch; **5** left common carotid artery; **6** brachiocephalic trunk; **7** right common carotid artery; **8** right subclavian artery; **9** thyroid ima artery;

**Fig. 2:**
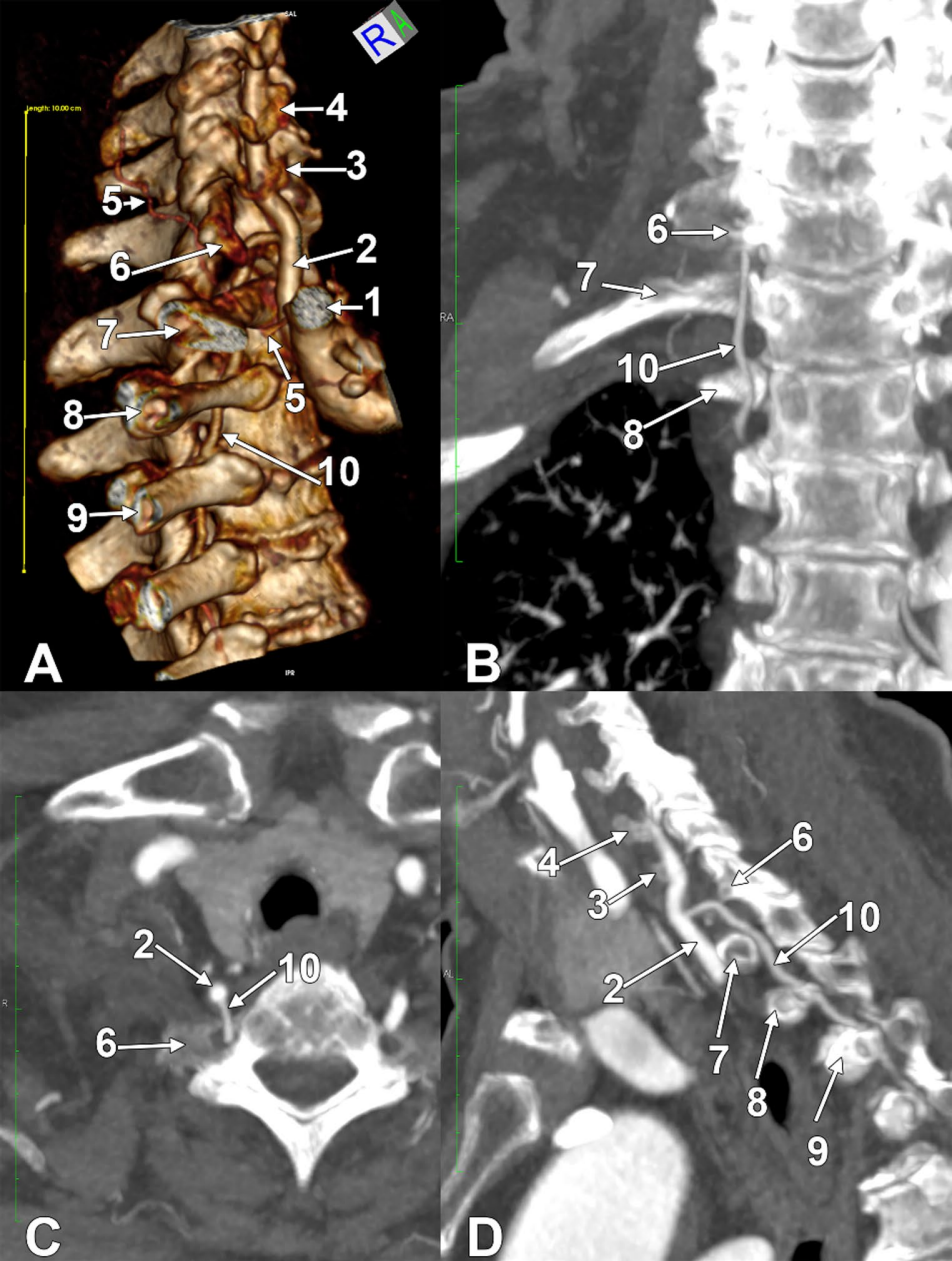
The variant right supreme intercostal artery (SIA). **A** Three-dimensional rendering, right-infero-lateral view. **B** Coronal slice through the SIA, anterior view. **C** Axial slice through the SIA, inferior view. **D** Sagittal slice through the SIA, medial view. **1** right subclavian artery; **2** vertebral artery; **3** C6 transverse process; **4** C5 transverse process; **5** deep cervical artery; **6** C7 transverse process; **7** first rib; **8** s rib; **9** third rib; **10** SIA (variant)

The variants are depicted in Figs. 1 and 2 and summarised in Table 1.

**Table 1:** Summary of concurrent variants.

Variant	Key measurements	Anatomical consequence	Clinical implication
Bovine aortic arch (common BCT–L-CCA origin) [Vascular variant 1]	Common trunk 2.03 × 0.94 cm; origin T1–T2	BCT originates left of midline; left-to-right tracheal course	Expanded anterior tracheal contact zone [9]
High pretracheal BCT course [Vascular variant 2]	Calibre 1.29 cm; upper margin 1.74 cm above manubrial notch (below the 2 cm Cai threshold)	Crosses anterior trachea L → R above manubrium	Haemorrhage risk: tracheotomy, thyroidectomy [3, 9]
TIA from BCT [Vascular variant 3]	0.49 cm from R lobe; 0.77 cm from isthmus	Bilateral distribution; loops on BCT anterior surface	Unrecognised TIA retracts to mediastinum if divided [2, 11]
RSA inferior loop + intrathoracic right VA origin [Vascular variant 4]	RSA loop 1.72 cm; VA origin 0.99 cm below rib 1 neck	Right VA initial segment intrathoracic (pulmonary apex medial)	Right VA access carries pleural entry risk [6]
SIA / TVA from right VA at C7 [Vascular variant 5]	Calibre 1.1 mm; initial segment 8.9 mm; posterior to rib necks 1–3	Descending TVA configuration through C7 transverse foramen	VA intervention may affect intercostal/bronchial supply[1, 5, 6, 7]
Poor SCJ articulation (bilateral) [Skeletal variant]	< 25% cortical contact; thyroid lobes posterior to clavicular heads	Suprasternal notch becomes interclavicular space	BCT + TIA + isthmus occupy single interclavicular pretracheal plane [9]

The table lists five vascular variants (variants 1–5) and one skeletal variant, consistent with the text. The right-hand entries in the clinical-implication column give the corresponding literature references

BCT, brachiocephalic trunk; TIA , thyroid ima artery; RSA , right subclavian artery; VA, vertebral artery; SIA, supreme intercostal artery; TVA, horacic vertebral artery; SCJ, sternoclavicular joint

## Discussion

This case shows five concurrent vascular variants together with a bilateral skeletal anomaly in a single individual. Three findings, namely the high pretracheal BCT course, the TIA arising from it, and the SIA in TVA configuration, each have direct surgical relevance. Their co-occurrence, together with the modified suprasternal topography created by poor SCJ articulation, has not, to our knowledge and within the literature reviewed for this report, been described as a combined pattern.

The TIA is present in approximately 3.3–3.8% of adults [11] and arises most commonly from the BCT (~ 74% of TIA cases). When present, it runs anterior to the trachea and may be the sole inferior thyroid supply if both inferior thyroid arteries are hypoplastic or absent [2]. In the current case the TIA displayed bilateral distribution, with its main vessel reaching the left inferior pole and a subsidiary branch supplying the right lobe and isthmus. A high-lying BCT, defined by Cai et al. [3] by an upper margin 2 cm or more above the suprasternal notch (or a course above the sixth tracheal cartilage) and found in approximately 2.2% of patients undergoing anterior cervical tracheal surgery, independently places a high-pressure vessel anterior to the lower cervical trachea. In the present case the upper margin lay 1.74 cm above the manubrial notch, just short of this threshold, so the vessel is best described as following a high pretracheal course rather than meeting the formal high-riding criterion. Tsakotos et al. (2023) documented BCT variants with a high-riding course causing tracheal displacement and noted that recognition relies on preoperative CT [9]. When these two variants co-occur, as in the present case, both a major trunk and an accessory vessel occupy the same pretracheal surgical plane, which may increase haemorrhage risk during tracheotomy, thyroidectomy, and cricothyrotomy.

The SIA in TVA configuration is the rarest arterial finding in this case. Bergman et al. [1] note that 'this artery may be a branch of the vertebral, in which case it passes through the transverse foramen of the seventh cervical vertebra and between the ribs and transverse processes of the upper three intercostal spaces’, a description that matches the course documented on the present CTA. The embryological basis, as discussed by Gailloud (2014), involves retention of the C7 intersegmental artery's connection with the cervical vertebral chain [5]. The seventh cervical intersegmental artery is normally incorporated into the supreme intercostal artery, whereas persistence of its anastomosis with the vertebral chain rather than consolidation into the costocervical trunk explains this variant. Gailloud et al. [6] documented nine cases of descending TVA in an interventional neuroradiology series and emphasised the risk of spinal cord ischaemia during vertebral artery intervention if such variants are unrecognised. The case of Hsu et al. [7] is the only directly analogous imaging report identified in the literature. In the present case, the right VA had an additional anomaly, an intrathoracic initial segment from the RSA inferior loop. Endovascular access to the right VA would therefore traverse the mediastinum before reaching the SIA origin at C7.

The bilateral poor SCJ articulation (Tuscano et al. [10], prevalence 4.8% in asymptomatic patients), although a recognised normal variant of no independent surgical consequence [10], altered the surgical topography by converting the suprasternal notch into an interclavicular space containing the BCT, TIA, and thyroid isthmus. Any anterior midline approach, including tracheotomy or thyroid isthmectomy, would encounter all three structures within the same compressed interclavicular pretracheal plane before reaching the trachea.

Embryologically, the whole cluster can be read as a single, coordinated remodelling defect of the right cervicothoracic intersegmental arterial system rather than as a set of unrelated coincidences. During early development the dorsal aortae are connected to a longitudinal post-costal anastomosis by paired intersegmental arteries; the cervical intersegmental arteries regress except for the seventh, which contributes to the subclavian artery, while the longitudinal anastomoses between the upper six fuse to form the vertebral artery. The seventh cervical (C7) intersegmental artery is normally incorporated into the costocervical trunk and SIA. Persistence of its anastomosis with the longitudinal vertebral chain, rather than consolidation into the costocervical trunk, produces a SIA arising from the vertebral artery in the descending TVA configuration, following the intersegmental nomenclature validated angiographically by Gailloud [5]. In parallel, the right subclavian and vertebral segments retained a low, looped configuration, giving the inferior mediastinal RSA loop and the intrathoracic origin of the right vertebral artery. On the ventral aspect, the midline ventral pharyngeal/thyroid arterial plexus, which normally regresses once the bilateral superior and inferior thyroid arteries are established, persisted as the TIA arising from the BCT. The bovine-type common origin of the BCT and left common carotid artery reflects altered partitioning of the aortic sac. Thus the same developmental window—remodelling of the aortic arches, the cervical intersegmental arteries, and the ventral thyroid plexus—can account for each component of this case. A similar clustering of posterior thoracic wall variants, comprising a descending branch of the vertebral artery supplying three intercostal spaces alongside five posterior intercostal artery common trunks, was documented by Fanselow et al. [4]. More broadly, the subclavian–vertebral–thyroid arterial axis shows substantial co-variation: Kotil et al. [8] reported a unilateral thyrovertebral trunk in which the vertebral and inferior thyroid arteries arose from a single subclavian stem, illustrating the shared developmental derivation of the vertebral and thyroid vessels from the same intersegmental network. Together, these observations indicate that cervicothoracic arterial variations may occur in complex clusters with important implications for thyroid surgery, tracheotomy, vascular interventions, and CTA-based preoperative assessment [8]. The present case shows that multi-variant cervicothoracic arterial patterns may also involve the anterior cervical vasculature.

Preoperative CTA with three-dimensional reconstruction is well suited to identify this configuration before elective anterior neck or airway procedures. Detection of one component of this cluster, such as a high or high-riding BCT course, an anomalous vertebral artery origin, or poor SCJ articulation, should alert the radiologist or surgeon to inspect the remaining cervicothoracic arterial anatomy.

## Conclusion

Five concurrent cervical vascular variants plus bilateral poor SCJ articulation were identified on CTA in a 74-year-old female, including a TIA from a high-coursing BCT with bilateral thyroid distribution, an SIA in TVA configuration from the right vertebral artery at C7, and an intrathoracic initial segment of the right VA. The arterial variants can be interpreted within developmental remodelling of the cervicothoracic arteries and, in combination with the SCJ morphology, may add to anterior cervical operative risk and warrant awareness during planning. To our knowledge, and within the literature reviewed for this report, this particular combination has not previously been reported, although the inference rests on a single case and should be interpreted with appropriate caution.

## Data Availability

Research data can be obtained from the author upon reasonable request.
